# Influenza A(H3N2) subclade K (J.2.4.1) viruses associated with a surge at a university health clinic, Arizona, the United States, November to early December 2025

**DOI:** 10.2807/1560-7917.ES.2026.31.7.2600111

**Published:** 2026-02-19

**Authors:** Matthew Scotch, Temitope OC Faleye, Angelica Urquidez-Negrete, Bradley Bobbett, Veronica Boyle, Kelly Conard, Lucy Sublasky-Rodriguez, Vel Murugan

**Affiliations:** 1 Arizona State University, Tempe, Arizona, the United States

**Keywords:** Influenza A Virus, H3N2 Subtype, Molecular Epidemiology, Universities, Evolution, Molecular, Public Health Surveillance

## Abstract

Genomic surveillance during an influenza surge between November and early December 2025 at a university health clinic in the United States identified A(H3N2) subclade K (J.2.4.1) viruses with shared haemagglutinin amino acid substitutions in antigenic sites and the receptor-binding domain. An epitope-based model indicated reduced vaccine protection (mean predicted protection 0.13). Phylodynamic analyses suggested multiple introductions with onward campus-to-community spread, highlighting that universities and other semi-closed settings can amplify transmission and aid early characterisation of emerging lineages.

In November 2025, we observed a marked increase in influenza diagnoses at a United States (US) university student health clinic. In response to concerns about A(H3N2) subclade K (J.2.4.1), we sequenced positive swab specimens collected during the surge and conducted genomic characterisation and phylodynamic analyses. We aimed to determine whether the surge was associated with subclade K and to assess potential antigenic and transmission-related implications in a university-associated population with frequent campus-community mixing.

## Detection of influenza in university students

We collected nasopharyngeal swabs from consenting participants with influenza-like illness who presented to a university student health clinic on a large urban campus (55,000 students [1]) in Tempe, Arizona, US, during the 2025/26 northern hemisphere influenza season. We recorded demographic information, antigen test results, vaccination history and other clinical data as part of the university’s involvement with the Centers for Disease Control and Prevention (CDC) US Flu Vaccine Effectiveness (VE) Network [2]. We performed RT-qPCR to confirm and subtype samples positive for influenza virus. After an unusual spike in cases in November, we further investigated 27 samples collected between 5 November and 2 December 2025 and sent them to the CDC for high-throughput sequencing of all eight influenza A gene segments. We provide the weekly counts of influenza diagnoses at the clinic and percent positivity in Supplementary Figure 1. The median age of the participants was 19 years (interquartile range (IQR): 18–19), and four reported receiving the 2025/26 influenza vaccine, while 23 reported no influenza vaccination. To support data sharing, CDC staff submitted the consensus sequences to GISAID (https://gisaid.org/), as presented in Supplementary Table 1.

## Genomic characterisation

We used Nextclade [3] to classify coding-complete haemagglutinin (HA) gene sequences (1,701 nt), which assigned all viruses to subclade K, as presented in Supplementary Figure 2A. We calculated whole genome pairwise identity using sequence demarcation tool (SDT)2 [4], and found high similarity among the viruses, as demonstrated in Supplementary Figure 2B. We used FluSurver [5] to identify HA amino acid substitutions and pEpitope [6] to estimate predicted vaccine protection of our translated sequences relative to the 2025/26 northern hemisphere vaccine strain A/District of Columbia/27/2023 (EPI_ISL_18937823), which Nextclade assigns to clade J.2.

## Genomic epidemiology of subclade K

To explore spatiotemporal transmission dynamics, we assigned each virus to one of three location categories based on participant residence: Tempe campus, Tempe off-campus and outside Tempe, as presented in Supplementary Table 2. We also categorised participants by 2025/26 vaccination status (influenza vaccine, no influenza vaccine). We performed Bayesian phylodynamics to understand the evolutionary diffusion and characteristics of local transmission, as presented in Supplementary Methods.

**Figure 1:**
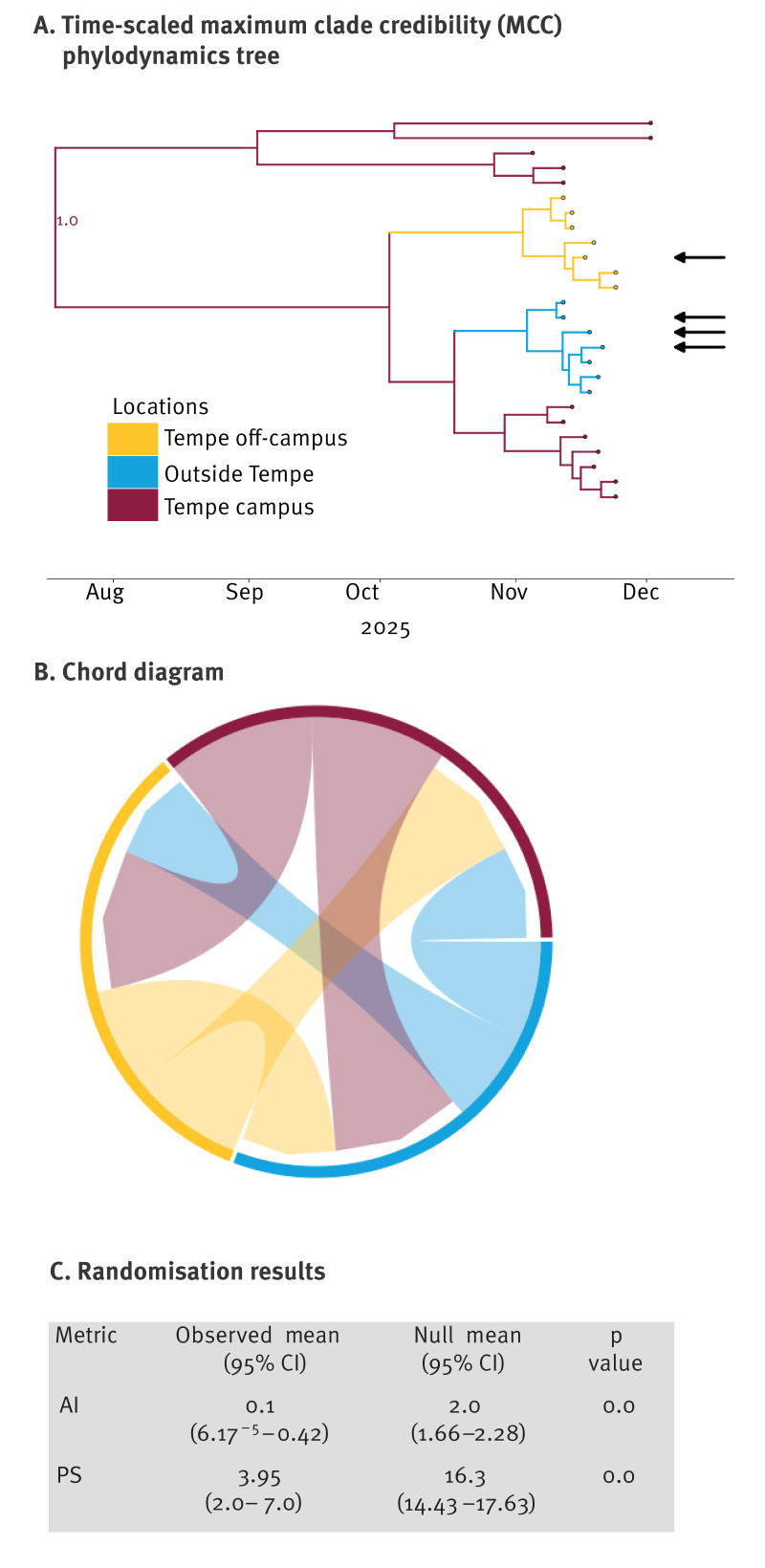
Phylodynamic analyses of haemagglutinin sequences of influenza A(H3N2) subclade K (J.2.4.1) virus with location metadata, Tempe, Arizona, the United States, November–early December 2025 (n = 26)

AI: association index; BaTS: Bayesian analysis of Tip Significance; PS: parsimony score.

The expansion of subclade K occurred near 19 July 2025 (Bayesian Highest Posterior Density 11 April 2025–28 September 2025). Figure 1A shows a time-scaled maximum clade credibility (MCC) phylodynamic tree, revealing early predominance of these viruses on campus followed by transmission to off-campus and locations outside the city. The chord diagram (Figure 1B), quantified by Markov jumps, indicates a relative balance in source-sink transmission dynamics. The tip-based association test results from the Bayesian analysis of Tip Significance (BaTS) programme [7] (Figure 1C) shows statistical significance (p < 0.01 for 100 randomisations) for both the association index (AI) and parsimony score (PS), indicating that the phylogeny of the K subclade was structured by student residence.

The phylodynamic analyses included 26 of 27 participants with residence metadata.

Panel A. Maximum clade credibility time-resolved haemagglutinin phylogeny with ancestral state reconstruction of student residence. Branch colours indicate the most probably inferred location state (Tempe campus, Tempe off-campus, or outside Tempe). Posterior probability is shown for the location state of the root node. Arrows indicate the taxa associated with a 2025/26 influenza vaccination. The Baltic library in Python [12] was used for tree visualisation.

Panel B. Chord diagram of our Markov jumps of the location state changes with the colours matching the branch colours in Panel A.

Panel C. BaTS analysis of the location trait among the posterior set of trees from the Markov chain Monte-Carlo simulation.

## Amino acid substitutions

**Figure 2:**
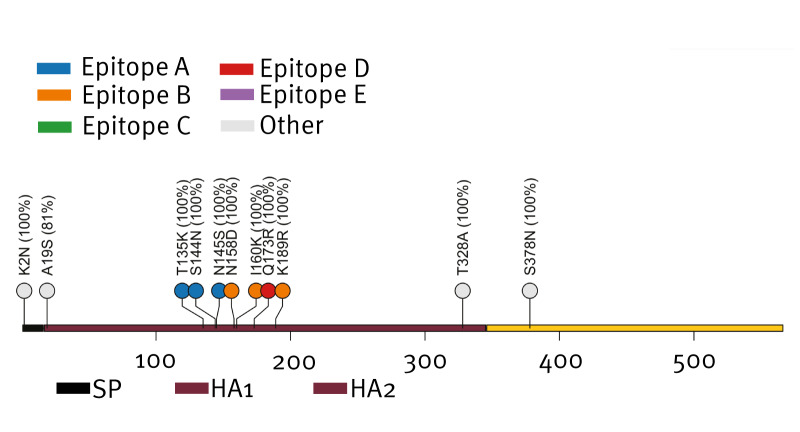
Amino acid substitutions relative to the 2025/26 North American H3N2 cell-based influenza vaccine strain A/District of Columbia/27/2023 (EPI_ISL_18937823), Tempe, Arizona, the United States, November–early December 2025 (n = 27)

HA: haemagglutinin; SP: signal peptide.

We observed 10 common subclade K amino acid substitutions [8] present in  ≥ 80% (26/27) of our translated haemagglutinin sequences including K2N, T135K, S144N, N145S, N158D, I160K, Q173R, K189R, T328A and S378N. Eight of these substitutions occurred in the HA1 subunit, seven of which mapped to known antigenic sites A, B, and D (Figure 2) and the receptor-binding domain (RBD).

The colours of the lollipops represent the known epitope regions (or other) with percentages indicating their proportion among the 27 university sequences. We show the substitutions with a proportion of ≥ 80%.

## Predicted vaccine protection

To assess the antigenic implications of these substitutions, we applied a sequence-based epitope model that predicted reduced protection of A/District of Columbia/27/2023. Among 27 university HA sequences, 26 showed a predicted VE of 0.13 (standard error (SE): 0.12) with epitope A dominance. The results are presented in Supplementary Table 3. One sequence had markedly lower predicted vaccine protection (0.02; SE: 0.13) with epitope B dominance and carried HA1 T127A, which FluSurver [5] indicates may remove a potential N-linked glycosylation site (NWT → NWA) with possible antigenic implications.

## Genetic structuring of host vaccination

We further evaluated whether viral genetic relatedness correlated with host vaccination status among our 27 university samples. We found a statistically significant difference for association index and parsimony score (p value < 0.01; 100 randomisations), indicating evidence of non-random phylogenetic distribution of host vaccination status, as presented in Supplementary Table 4.

## Discussion

We investigated an influenza surge at a university health clinic in Arizona in November–early December 2025 and found that all 27 haemagglutinin sequences included belonged to subclade K (J.2.4.1) and shared substitutions in recognised antigenic sites and the RBD [8]. A sequence-based epitope model predicted reduced protection against the 2025/26 cell-based vaccine reference strain (mean VE: 0.13), consistent with antigenic drift. Published interim VE estimates for the 2025/26 northern hemisphere season remain limited. A European Union cohort reported an A(H3N2) VE of 0.52 (95% confidence interval (CI): 0.29–0.69) [9], while a study in England reported results of 0.75 (95% CI: 0.52–0.88), 0.6 (95% CI: 0.15–0.85), and 0.35 (95% CI: −0.09–0.63) for A(H3N2) VE against emergency department attendance for persons aged 2–17, 18–64 and ≥ 65 years, respectively [10]. The authors found similar results for protection against hospital admission. Meanwhile, a study in Canada reported subclade K-specific VE estimates of 0.37 (95% CI: 0.20–0.50) [11].

To place these antigenic findings in a local transmission context, we examined the evolutionary diffusion of subclade K within the university community. The MCC tree summarises the ancestral state reconstruction of the three location states across the sampled HA sequences, with the root inferred to be campus-associated (posterior probability: 1.0). Analyses of the posterior distribution of phylodynamic trees revealed little support for a single campus-associated transmission lineage. Sequences sampled from 12 campus residents formed a monophyletic clade in only 0.3% of posterior trees (95% CI: 0.2–0.3%), indicating that campus-associated viruses were highly polyphyletic. In contrast, sequences sampled from Tempe off campus (n = 7) and from outside Tempe (n = 7) were more monophyletic in 51.3% (95% CI: 50.6–52.1%) and 56.5% (95% CI: 55.8–57.2%) of trees, respectively, reflecting moderate posterior support for clustering. Despite limited support for strict monophyly, BaTS detected significant phylogenetic association by location (p < 0.01), indicating non-random spatial structure across the posterior tree set. Together, these findings suggest that the university campus functioned primarily as a site of repeated viral introductions and mixing rather than a single sustained transmission chain.

This analysis has limitations. Sampling was restricted to symptomatic clinic visitors enrolled in the US Flu VE Network study, and sequencing captured only a snapshot of positives (n = 27). As a result, these data may not reflect the full viral diversity circulating in the study population. Phylodynamic analyses included aggregated location states due to sampling imbalance, potentially obscuring finer geographical structure. Associations related to vaccination status should be interpreted cautiously due to the imbalance between vaccinated and unvaccinated participants.

## Conclusion

The influenza surge observed at a US university health clinic was associated with A(H3N2) subclade K (J.2.4.1) viruses carrying shared haemagglutinin substitutions in antigenic sites and the receptor-binding domain. While the epitope-based results provide a sequence-derived signal of reduced vaccine protection, interim test-negative estimates demonstrate measurable protection against subclade K and underscore the importance of interpreting genomic signals within an immuno-epidemiological framework. These findings highlight the value of timely genomic surveillance in universities and other semi-closed settings to support rapid strain monitoring as subclade K continues to circulate.

## Data Availability

We submitted our sequences to GISAID that have been assigned the following accession numbers: EPI_ISL_20289461, EPI_ISL_20285045, EPI_ISL_20289460, EPI_ISL_20289463, EPI_ISL_20285047, EPI_ISL_20289462, EPI_ISL_20289457, EPI_ISL_20289459, EPI_ISL_20285043, EPI_ISL_20289458, EPI_ISL_20285053, EPI_ISL_20285055, EPI_ISL_20289465, EPI_ISL_20285049, EPI_ISL_20289464, EPI_ISL_20285051, EPI_ISL_20285077, EPI_ISL_20285079, EPI_ISL_20285073, EPI_ISL_20285075, EPI_ISL_20285061, EPI_ISL_20285057, EPI_ISL_20285059, EPI_ISL_20285069, EPI_ISL_20285071, EPI_ISL_20285065, EPI_ISL_20285067.

## Supplementary Data

484000 Supplementary Material
