# Supplementary material for: Influenza A(H3N2) subclade K (J.2.4.1) viruses associated with a surge at a university health clinic, Arizona, the United States, November to early December 2025
Source: Euro Surveill. 2026 Feb 19;31(7):2600111. doi: 10.2807/1560-7917.ES.2026.31.7.2600111 (PMC12924002; doi:10.2807/1560-7917.ES.2026.31.7.2600111)
Supplement: Supplementary Material [file 26-00111_SCOTCH_Supplement.pdf]

Supplementary Material

This supplementary material is hosted by *Eurosurveillance* as supporting information alongside the article **Influenza A(H3N2) subclade K (J.2.4.1) viruses associated with a surge at a university health clinic, Arizona, the United States, November–early December 2025**, on behalf of the authors, who remain responsible for the accuracy and appropriateness of the content. The same standards for ethics, copyright, attributions and permissions as for the article apply. Supplements are not edited by *Eurosurveillance,* and the journal is not responsible for the maintenance of any links or email addresses provided therein.

Supplementary Methods

Sample Collection, Processing, and Sequencing

We collected samples from individuals with influenza-like illness (ILI) who presented to the student health clinic at Arizona State University (ASU) from September 2025 to early January 2026 and consented to participate in the CDC US Flu Vaccine Effectiveness (VE) Network study [1]. The clinical staff collected nasopharyngeal (NP) swabs and tested for influenza via a rapid antigen-based test that assigns samples as influenza: *A positive*, *B positive*, *both A and B positive*, and *negative*. We transferred samples to the Biodesign Institute (ASU), tested them by RT-qPCR to confirm influenza virus positivity, and then ran separate RT-qPCR assays to subtype influenza A-positive samples. We reported Ct values and patient metadata via the CDC US Flu VE REDCap database. We sent samples to the CDC's Influenza Division for high-throughput Illumina sequencing. For each sample, CDC staff generated consensus sequences for all eight influenza gene segments and deposited them in GISAID.

Sample Metadata

We linked each sequence to patient metadata including census tract of residence and 2025–2026 vaccination status. We used the 2020 Census Tract Identifier [2] to group each tract into three location categories, including *Tempe campus*, *Tempe off-campus*, *outside Tempe*. We designated a tract as *Tempe campus* when a large portion of its area corresponded to the known ASU Tempe campus footprint. Because census tracts are heterogeneous, some university facilities fall inside tracts classified as *Tempe off-campus*. For one sample, we were unable to identify a census tract code.

Sequence Quality, Clade Assignment, and Whole-Genome Similarity

We uploaded our 27 hemagglutinin (HA) sequences to the Nextclade [3] web browser for quality assessment and clade assignment. All HA sequences received a quality score of *good* and an assignment of *K* with reference sequence A/Darwin/6/2021 (EPI_ISL_1563628). We analyzed the Nextclade-produced phylogenetic tree to compare our viruses with related GISAID sequences. To quantify genetic similarity among sampled viruses, we concatenated the eight influenza A gene segments of each virus in Geneious Prime v2025.2.2 [4], and computed pairwise nucleotide similarity using SDT2 [5].

Amino Acid Substitutions

We used Geneious Prime v2025.2.2 [4] to translate our hemagglutinin sequences and aligned them to the 2025–2026 vaccine reference strain *A/District Of Columbia/27/2023* (EPI_ISL_18937823) via Muscle v5.1 [6]. We used FluServer to confirm amino acid substitutions [7] and pEpitope [8] to estimate predicted vaccine protection relative to the reference strain.

Bayesian Inference

We implemented separate Bayesian inference models to analyze *location* and *vaccination status* as discrete traits. For the location analysis, we included 26 hemagglutinin (HA) sequences with associated location metadata, whereas for vaccination status we used all 27 HA

sequences in the study. We aligned sequences using MAFFT [9] through the *align* function in augur [10].

We performed Bayesian phylogenetic inference using BEAST v1.10 [11] by running two independent Markov chain Monte-Carlo simulations under a strict molecular clock and an exponential population growth prior [12]. For each trait, we specified a Bayesian stochastic search variable selection (BSSVS) process with an asymmetric discrete trait transition rate matrix. We recorded the transitions among discrete states using Markov jump counts [13] to support downstream posterior phylodynamic analyses. We assessed convergence and mixing using Tracer v1.7 [14] and confirmed that all parameters achieved effective sampling sizes (ESS) ≥ 200. We combined posterior log files from independent runs using LogCombiner in BEAST v1.10 [11] by specifying a 10% burn-in and generated a maximum clade credibility (MCC) tree using TreeAnnotator [11].

Although the Bayesian inference framework estimates the evolutionary and trait transition processes, we required additional analyses to interpret the posterior distribution and to quantify trait-associated clustering and transition dynamics.

Posterior Analyses

We characterized patterns of trait association and state transition by analyzing the posterior distribution of phylodynamic trees. Specifically, we used the *TreeMarkovJumpHistoryAnalyzer* library in BEAST v1.10.5 to extract posterior summaries of Markov jump counts, which quantify the number of inferred transitions among discrete location states along branches in the posterior tree set.

To evaluate whether closely related taxa shared location or vaccination traits more often than expected by chance, we used BaTS v0.2 [15] to generate null distributions based on 100 random permutations of trait-labels. We applied this analysis separately to the posterior tree set annotated with location traits (n = 3) and to the posterior tree set annotated with vaccination status (n = 2).

We quantified location-based clustering by testing whether sequences from each location formed monophyletic clades across the posterior distribution of trees. For each tree in our posterior set, we tested whether all observed sequences associated with a given location formed a monophyletic clade using the *is.monophyletic* function in the R package *ape* [16]. We summarized support for clustering as the proportion of posterior trees in which each group was monophyletic and estimated 95% confidence intervals using binomial tests.

References

1. CDC. US Flu VE Network 2025 [Available from: https://www.cdc.gov/flu-vaccines-work/php/vaccine-effectiveness/us-flu-ve-network.html.
2. US Census Bureau. 2020 Census Tract Identifier 2026 [Available from: https://mtgis-portal.geo.census.gov/arcgis/apps/experiencebuilder/experience/?id=bc7d5cafd5e94dfb875ac36df0deaf77.
3. Aksamentov I, Roemer C, Hodcroft EB, Neher RA. Nextclade: clade assignment, mutation calling and quality control for viral genomes. Journal of Open Source Software. 2021;6(67):3773.
4. Kearse M, Moir R, Wilson A, Stones-Havas S, Cheung M, Sturrock S, et al. Geneious Basic: an integrated and extendable desktop software platform for the organization and analysis of sequence data. Bioinformatics. 2012;28(12):1647-9.
5. Muhire BM, Varsani A, Martin DP. SDT: a virus classification tool based on pairwise sequence alignment and identity calculation. PLoS One. 2014;9(9):e108277.
6. Edgar RC. Muscle5: High-accuracy alignment ensembles enable unbiased assessments of sequence homology and phylogeny. Nature Communications. 2022;13(1):6968.
7. Maurer-Stroh S, Lee RTC, Limviphuvadh V, Ma J, Sirota FL, Gunalan V, et al. FluServer: Real-time surveillance of influenza mutations 2025 [Available from: http://flusurver.bii.a-star.edu.sg.
8. Bonomo ME, Kim RY, Deem MW. Modular epitope binding predicts influenza quasispecies dominance and vaccine effectiveness: Application to 2018/19 season. Vaccine. 2019;37(24):3154-8.
9. Katoh K, Misawa K, Kuma K, Miyata T. MAFFT: a novel method for rapid multiple sequence alignment based on fast Fourier transform. Nucleic Acids Res. 2002;30(14):3059-66.
10. Huddleston J, Hadfield J, Sibley TR, Lee J, Fay K, Ilcisin M, et al. Augur: a bioinformatics toolkit for phylogenetic analyses of human pathogens. J Open Source Softw. 2021;6(57).
11. Suchard MA, Lemey P, Baele G, Ayres DL, Drummond AJ, Rambaut A. Bayesian phylogenetic and phylodynamic data integration using BEAST 1.10. Virus Evol. 2018;4(1):vey016.
12. Griffiths RC, Tavare S. Sampling theory for neutral alleles in a varying environment. Philos Trans R Soc Lond B Biol Sci. 1994;344(1310):403-10.
13. Minin VN, Suchard MA. Counting labeled transitions in continuous-time Markov models of evolution. J Math Biol. 2008;56(3):391-412.
14. Rambaut A, Drummond AJ, Xie D, Baele G, Suchard MA. Posterior Summarization in Bayesian Phylogenetics Using Tracer 1.7. Syst Biol. 2018;67(5):901-4.
15. Parker J, Rambaut A, Pybus OG. Correlating viral phenotypes with phylogeny: accounting for phylogenetic uncertainty. Infect Genet Evol. 2008;8(3):239-46.
16. Paradis E, Schliep K. ape 5.0: an environment for modern phylogenetics and evolutionary analyses in R. Bioinformatics. 2019;35(3):526-8.

Tables

Supplementary Table 1. The 27 GISAID records produced from this study.

GISAID	Strain Name	Collection Date
EPI_ISL_20285067	A/Arizona/139/2025	2025-11-05
EPI_ISL_20285049	A/Arizona/140/2025	2025-11-12
EPI_ISL_20285059	A/Arizona/141/2025	2025-11-12
EPI_ISL_20285069	A/Arizona/142/2025	2025-11-12
EPI_ISL_20285079	A/Arizona/143/2025	2025-11-12
EPI_ISL_20285077	A/Arizona/144/2025	2025-11-12
EPI_ISL_20285043	A/Arizona/145/2025	2025-11-12
EPI_ISL_20285073	A/Arizona/146/2025	2025-11-14
EPI_ISL_20285047	A/Arizona/147/2025	2025-11-14
EPI_ISL_20285045	A/Arizona/148/2025	2025-11-14
EPI_ISL_20285065	A/Arizona/149/2025	2025-11-17
EPI_ISL_20285071	A/Arizona/150/2025	2025-11-17
EPI_ISL_20285051	A/Arizona/151/2025	2025-11-18
EPI_ISL_20285053	A/Arizona/152/2025	2025-11-18
EPI_ISL_20285055	A/Arizona/153/2025	2025-11-18
EPI_ISL_20285057	A/Arizona/154/2025	2025-11-19
EPI_ISL_20285061	A/Arizona/155/2025	2025-11-19
EPI_ISL_20285075	A/Arizona/156/2025	2025-11-20
EPI_ISL_20289461	A/Arizona/157/2025	2025-11-20
EPI_ISL_20289463	A/Arizona/158/2025	2025-11-21
EPI_ISL_20289459	A/Arizona/160/2025	2025-11-24
EPI_ISL_20289465	A/Arizona/161/2025	2025-11-24
EPI_ISL_20289460	A/Arizona/162/2025	2025-11-24
EPI_ISL_20289464	A/Arizona/163/2025	2025-11-24
EPI_ISL_20289457	A/Arizona/164/2025	2025-12-02
EPI_ISL_20289462	A/Arizona/165/2025	2025-12-02
EPI_ISL_20289458	A/Arizona/159/2025	2025-11-21

Supplementary Table 2. Census tract codes of the 26 study participants and their assigned locations for the phylodynamic model. We used the US Census Bureau's 2020 Census Tract Identifier [2] to map codes to locations. We were unable to link one sample to a census tract and thus it was not included in the model.

Census Tracts	Number of Participants	Assigned Location
04 013 3190 00<br>04 013 3187 00	12	Tempe campus
04 013 3191 01<br>04 013 3191 03<br>04 013 3191 04<br>04 013 3195 00<br>04 013 3197 05	7	Tempe off-campus
04 013 1050 03<br>04 013 1167 07<br>04 013 2168 49<br>04 013 6109 00<br>04 013 8161 00<br>04 013 8164 00<br>04 019 0046 14	7	outside Tempe
Unknown	1	not included

Supplementary Table 3. Predicted vaccine protection via pEpitope [8] for the 27 university samples against A/District Of Columbia/27/2023. We show the dominant epitope, predicted vaccine effectiveness (VE), its standard error (SE), and each amino acid substitution by epitope. We did not observe any substitutions in epitope E. We used FluServer [7] and their classical H3 HA numbering system.

Sequence	Dominant Epitope	Predicted VE	SE	Epitope<br>A	 B	 C	 D
EPI_ISL_20289461	A	0.13	0.12	T135K<br>S144N<br>N145S	N158D<br>I160K<br>K189R	none	Q173R
EPI_ISL_20285045	B	0.02	0.13	T135K<br>S144N<br>N145S	T127A<br>N158D<br>I160K<br>K189R	none	Q173R
EPI_ISL_20289460	A	0.13	0.12	T135K<br>S144N<br>N145S	N158D<br>I160K<br>K189R	none	Q173R
EPI_ISL_20289463	A	0.13	0.12	T135K<br>S144N<br>N145S	N158D<br>I160K<br>K189R	none	Q173R
EPI_ISL_20285047	A	0.13	0.12	T135K<br>S144N<br>N145S	N158D<br>I160K<br>K189R	none	Q173R
EPI_ISL_20289462	A	0.13	0.12	T135K<br>S144N<br>N145S	N158D<br>I160K<br>K189R	none	K171R<br>Q173R
EPI_ISL_20289457	A	0.13	0.12	T135K<br>S144N<br>N145S	N158D<br>I160K<br>K189R	K278E	Q173R
EPI_ISL_20289459	A	0.13	0.12	T135K<br>S144N<br>N145S	N158D<br>I160K<br>K189R	none	Q173R
EPI_ISL_20285043	A	0.13	0.12	T135K<br>S144N<br>N145S	N158D<br>I160K<br>K189R	none	Q173R
EPI_ISL_20289458	A	0.13	0.12	T135K<br>S144N<br>N145S	N158D<br>I160K<br>K189R	none	Q173R
EPI_ISL_20285053	A	0.13	0.12	T135K<br>S144N<br>N145S	N158D<br>I160K<br>K189R	none	Q173R
EPI_ISL_20285055	A	0.13	0.12	T135K<br>S144N<br>N145S	N158D<br>I160K<br>K189R	none	Q173R
EPI_ISL_20289465	A	0.13	0.12	T135K<br>S144N<br>N145S	N158D<br>I160K<br>K189R	none	Q173R
EPI_ISL_20285049	A	0.13	0.12	T135K<br>S144N<br>N145S	N158D<br>I160K<br>K189R	none	Q173R
EPI_ISL_20289464	A	0.13	0.12	T135K<br>S144N<br>N145S	N158D<br>I160K<br>K189R	none	Q173R
EPI_ISL_20285051	A	0.13	0.12	T135K<br>S144N<br>N145S	N158D<br>I160K<br>K189R	none	Q173R
EPI_ISL_20285077	A	0.13	0.12	T135K<br>S144N<br>N145S	N158D<br>I160K<br>K189R	none	Q173R

EPI_ISL_20285079	A	0.13	0.12	T135K<br>S144N<br>N145S	N158D<br>I160K<br>K189R	none	Q173R
EPI_ISL_20285073	A	0.13	0.12	T135K<br>S144N<br>N145S	N158D<br>I160K<br>K189R	none	Q173R
EPI_ISL_20285075	A	0.13	0.12	T135K<br>S144N<br>N145S	N158D<br>I160K<br>K189R	none	Q173R
EPI_ISL_20285061	A	0.13	0.12	T135K<br>S144N<br>N145S	N158D<br>I160K<br>K189R	none	Q173R
EPI_ISL_20285057	A	0.13	0.12	T135K<br>S144N<br>N145S	N158D<br>I160K<br>K189R	none	Q173R
EPI_ISL_20285059	A	0.13	0.12	T135K<br>S144N<br>N145S	N158D<br>I160K<br>K189R	none	Q173R
EPI_ISL_20285069	A	0.13	0.12	T135K<br>S144N<br>N145S	N158D<br>I160K<br>K189R	none	Q173R
EPI_ISL_20285071	A	0.13	0.12	T135K<br>S144N<br>N145S	N158D<br>I160K<br>K189R	none	Q173R
EPI_ISL_20285065	A	0.13	0.12	T135K<br>S144N<br>N145S	N158D<br>I160K<br>K189R	none	Q173R
EPI_ISL_20285067	A	0.13	0.12	T135K<br>S144N<br>N145S	N158D<br>I160K<br>K189R	none	Q173R

Supplementary Table 4. Bayesian tip significance results for vaccination status.
Abbreviations: CI – Confidence Interval.

Metric	Observed Mean (95% CI)	Null Mean (95% CI)	p-value
Association Index	0.06 (8.89^{-8}–0.35)	0.79 (0.6–0.97)	< 0.01
Parsimony Score	1.98 (1.0–4.0)	5.72 (5.3–5.95)	< 0.01

Figures

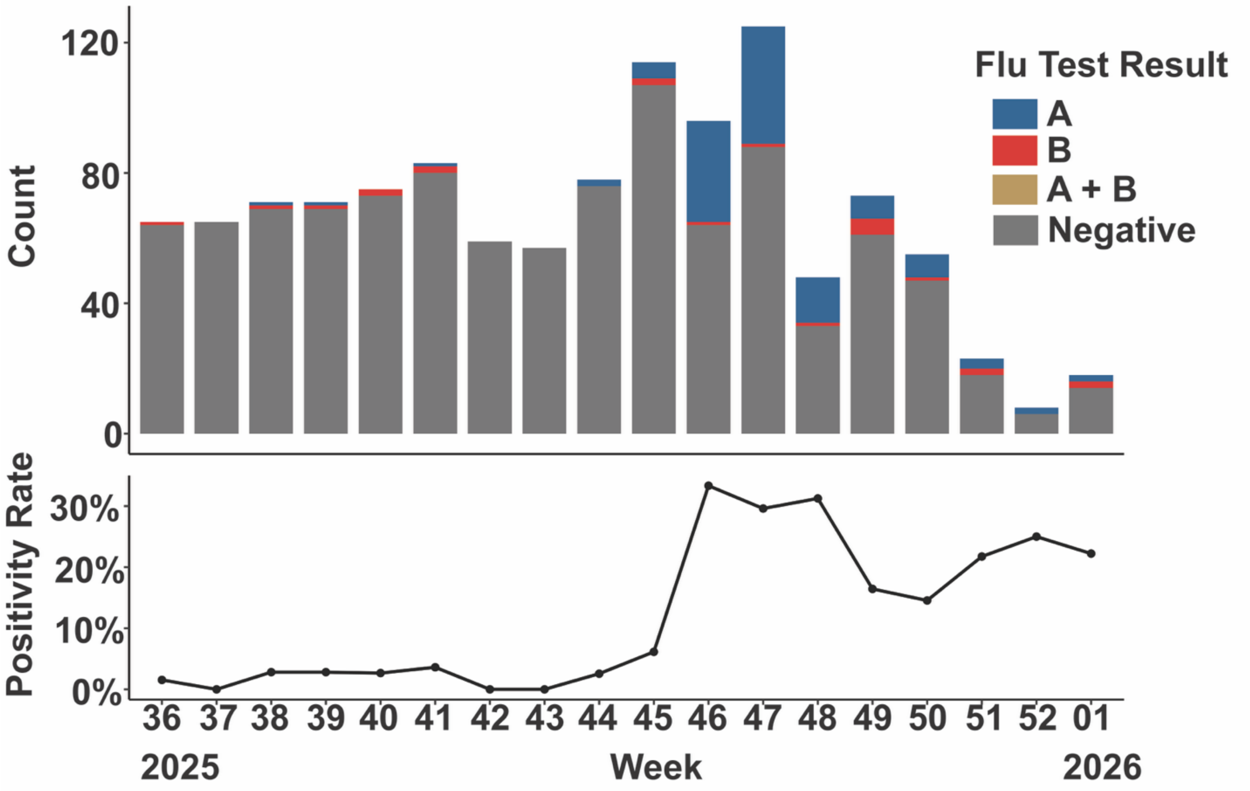

Supplementary Figure 1. Influenza test results at the university student health clinic. On the top, we show the antigen-based test results of the 1,184 nasopharyngeal (NP) swab samples collected between week 36 of 2025 to week 1 of 2026. On the bottom, we show the percent test positivity. During November (weeks 46 and 47), we observed a spike in cases and percent positivity likely due to subclade K.

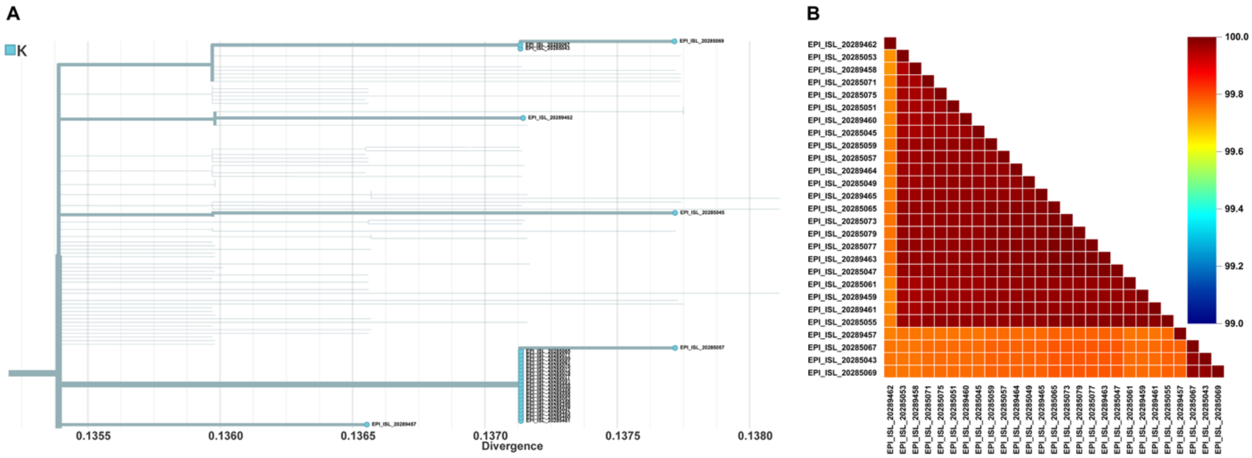

Supplementary Figure 2. Phylogeny and sequence similarity of the 27 university HA sequences. A) Phylogenetic tree generated by Nexclade [3] with a filter on the university sequences and grouped within subclade K of the larger tree. B) Whole-genome sequence similarity heatmap generated by SDT2 [5].
